# Medical Miscellany

**Published:** 1853-11

**Authors:** 


MEDICAL MISCELLANY.—A young woman was recently killed in New York City, it is stated, by the explosion of some extract of orange, while holding a lamp too near the can from which she was pouring it. Ought not the proprietor of the manufactory in which she was employed to have warned her of the danger? Ought not all persons who deal in or sell *dangerous liquids* of *all* kinds, solemnly and carefully to warn purchasers of the dangerous and sometimes fatal results of a use of the same?——Small pox rages fearfully at the Sandwich Islands, and is said to threaten extermination of the natives.——A German woman in Cincinnati lately gave birth to four children—two boys and two girls—all thriving at latest acccounts.——We have a number of times seen it announced in secular publications that camphor is an antidote to strychnine. It should be borne in mind, and at least a trial given it.——The American Vegetarian Association lately held a *meet*ing at Philadelphia. Grahamism ran loose, and one speaker declared that man had no more right to kill an animal than to kill a fellow man.——The Boston Med. and Surg. Journal says that a young gentleman lately died in Georgia, weighing 643 pounds: he died from excessive accumulation of fat.——An English paper states that a child was born in Exeter, Eng., with thirteen perfect fingers on the right hand.——The man who wrote a note to his family physician, stating that he had taken cold and "got a little horse," was advised by his Dr. to procure a saddle and bridle.——A child of Mr. Loren Field, of Conn., died lately from the effects of eating colored candy. Dr. Hood examined the contents of the stomach, and found enough lead to cause death.

